# M-CSF and IL-34 expression as indicators for growth in sporadic vestibular schwannoma

**DOI:** 10.1007/s00428-018-2503-1

**Published:** 2018-12-22

**Authors:** W. M. de Vries, I. H. Briaire-de Bruijn, P. P. G. van Benthem, A. G. L. van der Mey, P. C. W. Hogendoorn

**Affiliations:** 1 Department of Pathology, Leiden University Medical Center, P.O. Box 9600, 2300 RC Leiden, The Netherlands; 2 Department of Otolaryngology, Leiden University Medical Center, P.O. Box 9600, 2300 RC Leiden, The Netherlands

**Keywords:** IL-34, Macrophage colony stimulating factor, Vestibular schwannoma

## Abstract

Macrophage colony stimulating factor and IL-34 are associated with clinical vestibular schwannoma progression. Investigating the biology behind vestibular schwannoma progression helps understanding tumor growth. Inflammation is important in the microenvironment of neoplasms. Macrophages are major players in the intratumoral infiltrate. These tumor-associated macrophages are known to stimulate angiogenesis and cell growth. M-CSF and IL-34 are cytokines that can regulate tumor-infiltrating macrophages. They are expressed by tumors and form potential targets for therapy. The goal of this study was to investigate these cytokines in vestibular schwannomas and to see if their expression is related to angiogenesis, macrophage numbers, cystic degeneration, and volumetric tumor progression. Immunohistochemical expression of M-CSF and IL-34 was analyzed in ten fast-growing vestibular schwannomas and in ten slow-growing vestibular schwannomas. Expression M-CSF and IL-34 were compared between fast- versus slow-growing and cystic versus non-cystic tumors. Data on macrophage numbers and microvessel density, known from earlier research, was also included. All tumors expressed M-CSF and its expression was higher in fast-growing tumors (*p* = 0.003) and in cystic tumors (*p* = 0.035). CD163 expression was higher in tumors with strong M-CSF expression (*p* = 0.003). All tumors expressed IL-34 as well, but no significant differences were found in relation to clinicopathological characteristics. This study demonstrated the expression of M-CSF and IL-34 in vestibular schwannomas. The results suggest that M-CSF is related to macrophage activity and tumor progression, making it a potential target for therapy. If a similar assumption can be made for IL-34 remains unclear.

## Introduction

Vestibular schwannomas (VS) are benign neoplastic proliferations recapitulating the differentiation repertoire of the myelin-forming Schwann cells of the vestibular branch of the vestibulocochlear nerve in the internal auditory canal or the cerebellopontine angle. These tumors often display a slow and self-limiting growth pattern but there are also variants that progress more rapidly and persistently. In these patients, ongoing tumor progression can eventually cause brainstem compression or paralysis of adjacent cranial nerves. In most cases (> 90%), VS occur as unilateral sporadic tumors [1], whereas bilateral tumors are pathognomonic for the hereditary disorder neurofibromatosis type 2 (NF2) [2]. Loss of function of the tumor suppressor protein merlin, encoded by the *NF2* gene, is an essential step in schwannoma pathogenesis [3, 4]. Heterozygous germline inactivating mutations affecting the *NF2* gene cause neurofibromatosis type 2 while biallelic somatic mutations of *NF2* are found in sporadic VS [4]. Recent years showed an increase in the number of newly diagnosed VS to approximately 20 per million people per year [5–7]. This phenomenon most probably is the result of more frequent use of magnetic resonance imaging scanning (MRI), which in turn leads to the identification of more subclinical cases of VS.

Management of these VS comprises several options. The initial policy for smaller tumors is to wait and see by performing sequential MRI scans. In case of large tumors or when tumors rapidly progress active treatment is needed. Current therapeutic management of VS consists of microsurgery or radiotherapy. In selected cases of NF2-related tumors, pharmacotherapeutic options are also applied [8–10]. This kind of therapy is not used for sporadic VS. One of the clinical dilemmas in selecting the most suitable treatment policy for VS is the unpredictable behavior these tumors can display. Some tumors remain stable for decades while others double in size within less than a year. So far, cystic degeneration is the only known prognostic marker for progressive tumor volume growth [11]. Better prediction of tumor volume progression will improve the accuracy of determining the correct moment and modality of therapeutic intervention. More understanding of tumor behavior requires more insight into tumor biological factors influencing tumor development. Investigating VS biology not only benefits the understanding of its growth pattern but it will also contribute to the identification of potential therapeutic targets.

In two earlier papers on the inflammatory microenvironment in VS, we demonstrated a relationship between tumor-associated macrophages (TAM), angiogenesis, and tumor growth [12, 13]. These results were in line with the emerging notion that intratumoral inflammation is a major driving force behind the volumetric progression of tumors [14–16]. The fact that TAM may form a target for therapy emphasizes their potential clinical importance [17]. TAM consist of a heterogeneous population of, mainly alternatively activated, M2-type macrophages that seem to have tumor promoting characteristics [16, 18]. Inhibiting the formation of M2 macrophages may therefore have a negative effect on tumor progression. An important regulator within the inflammatory microenvironment capable of polarizing macrophages towards an M2-like phenotype is a cytokine known as the macrophage colony stimulating factor, or M-CSF [19]. The exact role of M-CSF in macrophage associated tumor development remains to be elucidated but its function as a promoter of tumor progression has been indicated in several tumor models [20–22]. Another regulating protein that seems to be capable of skewing the microenvironment into a tumor promoting direction is interleukin-34 (IL-34). This cytokine was first described in 2008 by Lin et al. [23] and displays common features with M-CSF in such a way that they appear to have synergistic functions [24, 25]. Consistent with these findings, relatively recent studies have indicated that IL-34 seems to be associated with tumor progression in osteosarcoma and lung cancer [26, 27].

The aim of this study was to analyze the expression of M-CSF and IL-34 in our earlier described cohort of 20 sporadic vestibular schwannoma patients and to determine whether their expression can be related to clinicopathologic characteristics and tumor growth.

## Materials and methods

We performed immunohistochemical stains against M-CSF and IL-34 on formalin-fixed paraffin-embedded vestibular schwannoma tissue. To investigate whether there is a relationship between the presence of these proteins and VS progression, we analyzed their expression pattern in the same selection of tumors that we previously analyzed and described for the expression of tumor-associated macrophages [12]. This selection of tumor samples consisted of ten radiologically observed fast-growing tumors and ten radiologically observed slower growing tumors. The expression patterns of M-CSF and IL-34 within these groups were compared with each other. The already published data on angiogenesis and macrophage expression was also included into this analysis, as well as radiological data regarding cystic degeneration.

### Patient selection

A retrospective selection was made from vestibular schwannoma database of the Leiden University Medical Center. This group of patients had been consecutively treated for a proven sporadic vestibular schwannoma from January 2006 to December 2011. Two different cohorts were compiled out of a total of 46 consecutively treated patients. All patients had at least two preoperative MRI scans on the basis of which volumetric tumor growth was measured. The first cohort included the ten patients with the slowest growing tumors, while the second cohort included the ten fastest growing tumors. The decision for surgical treatment had been based upon symptoms (e.g., vertigo, hearing loss, and tinnitus), tumor size, tumor growth, and patients’ personal preference. No NF2-related tumors were included in the analysis.

To ensure patients’ privacy, all samples were managed in a coded fashion and all procedures were conducted according to the Code for Proper Secondary Use of Human Tissue in the Netherlands (Dutch Federation of Medical Scientific Societies).

### Tumor measurement

**Fig. 1:**
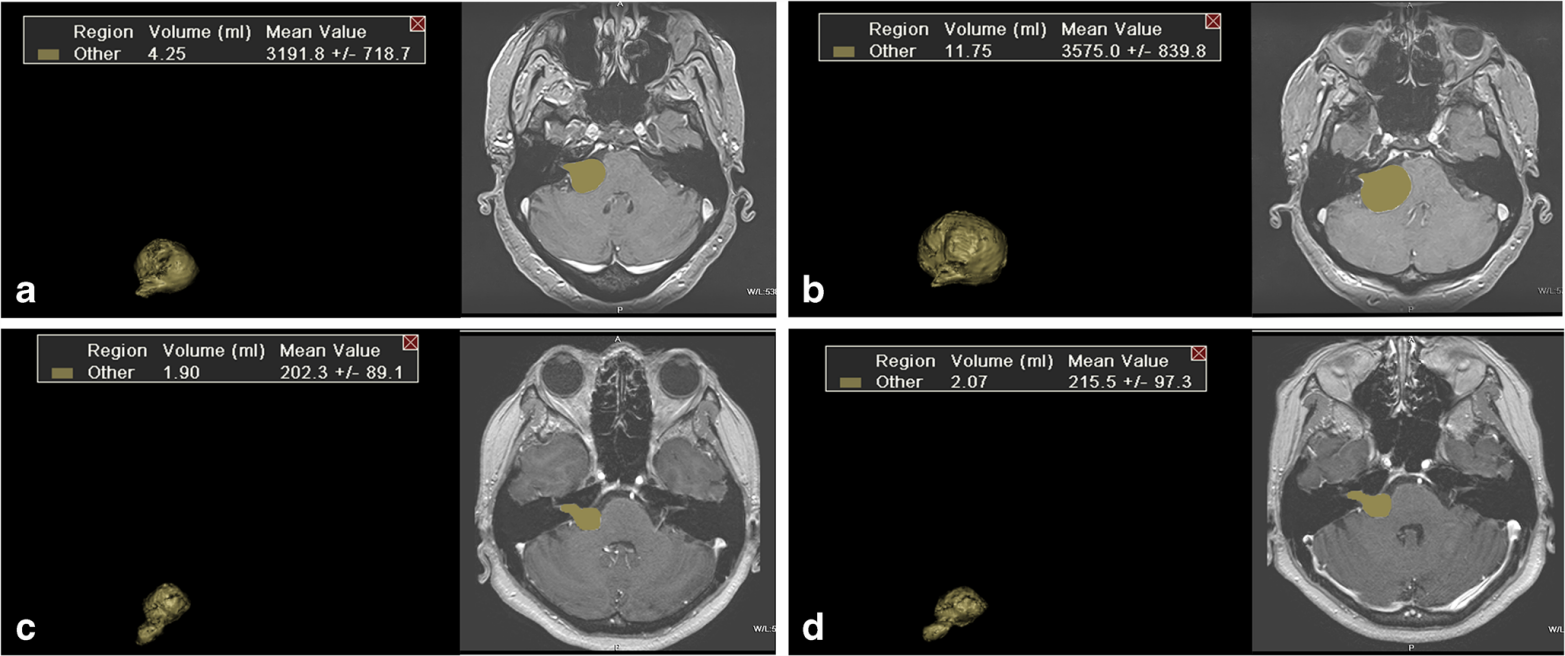
**a** and **b** A fast-growing tumor with an average growth of 9 ml per year. **c** and **d** A slower growing tumor with an average growth of 0.2 ml per year

The measurement of all tumors was performed on T1-weighted gadolinium-enhanced MRI scans and conducted by one and the same author. Tumor volume was determined with a contour measurement method using Vitrea View software (Vital Imaging, Minnetonka, MN, USA). To increase the accuracy of the measurements, each volume was determined two times per MRI scan. The mean of these two measurements was used for further evaluation of tumor growth. By calculating the difference in tumor volume on sequential MRI investigations, tumor growth rate could be determined. Volumetric growth was expressed as the increase in milliliters per year. Next to measuring tumor growth rate, the presence of cystic degeneration was evaluated. Figure 1 shows volume measurements of a fast-growing and a slow-growing tumor.

### Immunohistochemistry

Immunohistochemistry was performed on 4-μm thick slides acquired form formalin-fixed paraffin-embedded vestibular schwannoma tissue samples. All staining procedures were conducted on one and the same tumor block per tumor sample. The exact materials and methods that were applied for the CD31 and CD163 stains are described in the earlier mentioned paper on tumor-associated macrophages [12]. Immunohistochemical stains for M-CSF (pre-incubation and incubation in 5% non-fat milk in PBS/1%BSA, 1:100 diluted, anti-M-CSF antibody, ab52864, Abcam, Cambridge, MA, USA) and IL-34 (pre-incubation and incubation in 5% non-fat milk in PBS/1%BSA, 1:3000, anti-interleukin-34 antibody, ab224734, Abcam, Cambridge, MA, USA) were performed according to standard laboratory methods [28]. Tonsil served as positive control for M-CSF and hepatocellular carcinoma and prostate carcinoma were used as positive control tissues for IL-34.

### Microscopic analysis

Immunostainings were evaluated by two separate observers (W. de Vries and P. Hogendoorn) without knowledge of clinical patient data. M-CSF as well as IL-34 showed a varying staining pattern with areas of strong expression and areas of weak expression within the same tumor. Additionally, several tumor specimens showed hemosiderin deposition mimicking positive staining. This made computerized quantification less reliable. For this reason, a semiquantitative immunohistochemistry score of the overall staining intensity was made for each tumor sample. Staining intensity was initially categorized as no staining, weak staining, moderate staining, and strong staining. Exact details regarding M2 macrophage (CD163) and angiogenesis (CD31) staining and scoring techniques are described in our earlier report [12].

### Statistical analysis

The difference in M-CSF and IL-34 expression in fast- versus slow-growing tumors and cystic versus non-cystic tumors was determined with the Fisher’s exact test. The Mann-Whitney *U* test was used to determine the relation between the expression of M-CSF and IL-34 and the degree of microvessel density and CD163 expression. This test was also used to see if there was a relation between cystic degeneration and CD-163 expression. For all statistical tests a level of significance of *p* < 0.05 was taken into account. Calculations were made using SPSS version 16.0, IBM, Inc.

## Results

**Fig. 2:**
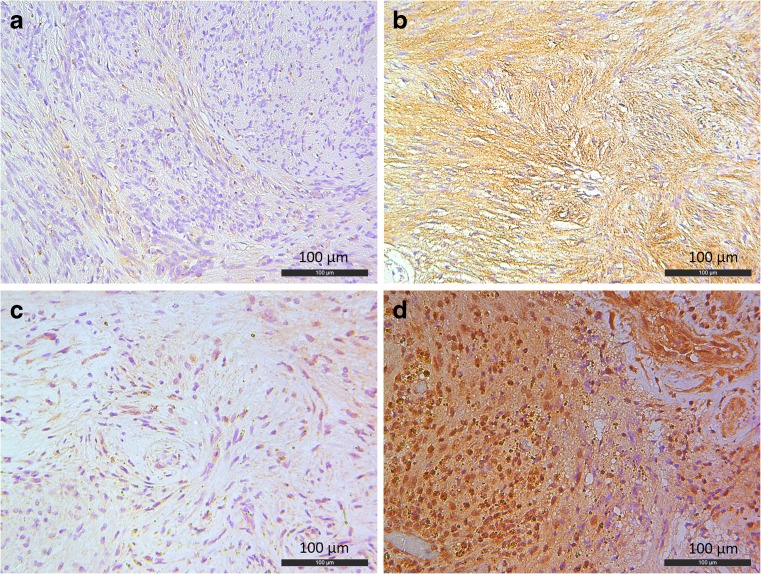
**a** Weak expression of M-CSF. **b** Strong expression of M-CSF. **c** A tissue area with weak IL-34 expression. **d** A tissue area with strong IL-34 expression

Details regarding patient characteristics are listed in Table 1. There were no significant differences in distribution of age, sex, and duration of preoperative follow-up between the two patient groups. As expected, the distribution of tumor growth rate and tumor volume as well as cystic degeneration was significantly different in the two groups. Analysis of the immunohistochemical stainings showed the following results. All samples were M-CSF positive, demonstrating mainly cytoplasmic expression in an irregular staining pattern (Fig. 2). Nine samples showed weak staining, four samples showed moderate staining, and seven samples showed strong staining. Based on these results, we simplified the categories of staining to weak and strong staining, the latter being the combination of moderate and strong staining. M-CSF staining was significantly higher in the group of fast-growing tumors (*p* = 0.003). M-CSF expression is also significantly higher (*p* = 0.035) in cystic tumors (Table 2). CD163 expression was significantly higher in tumors with strong M-CSF expression (*p* = 0.003). There was no significant relation between microvessel density and M-CSF expression.

**Table 1:** Patient characteristics

Case	Sex	Age (years)	Tumor volume (ml)	Growth rate (ml/year)	Preoperative MRI follow-up (months)	Cystic
Slow-growing group						
L3721	M	60	0.35	0.06	13	No
L3742	F	54	0.27	0.21	7	Yes
L3773	F	52	0.22	0.07	6	No
L3774	F	50	0.88	0.19	27	No
L3775	M	53	0.69	0.17	10	No
L3779	F	51	1.46	0.18	32	Yes
L3780	F	56	0.74	0.25	11	No
L3781	F	44	0.53	0.19	12	No
L3787	M	58	0.52	0.18	12	No
L3797	F	60	2.13	0.23	12	No
Mean (±SD)		53.8 (± 4.96)	0.77 (± 0.59)	0.17 (± 0.06)	14.2 (± 8.46)	
Fast-growing group						
L3725	F	62	3.26	1.33	11	No
L3731	F	81	14.41	4.41	19	Yes
L3733	F	76	11.66	8.84	10	No
L3740	F	52	2.27	2.50	7	No
L3741	F	71	7.25	1.83	21	Yes
L3745	F	56	5.62	2.18	6	Yes
L3746	M	46	30.73	43.98	4	Yes
L3792	F	75	6.92	3.43	21	Yes
L3793	F	39	7.32	5.52	10	Yes
L3805	F	39	23.05	2.90	9	Yes
Mean (±SD)		59.7 (± 15.65)	11.24 (± 9.16)	7.69 (± 12.94)	11.8 (± 6.26)	
Difference fast versus slow (*p*)	0.26a	0.41b	*< 0.0001b*	*< 0.0001b*	0.31b	*0.007a*

a, chi-square test; b, Mann-Whitney *U* test; results at *p* ≤ 0.05 are shown in italics

**Table 2:** Fisher’s exact test for M-CSF and IL-34 expression

	M-CSF staining		IL-34 staining	
	Weak	Strong	Moderate	Strong
Growth rate				
Slow	8	2	4	6
Fast	1	9	4	6
Total	9	11	8	12
*p* value		*0.003*		0.675
Cystic degeneration				
No	7	3	5	5
Yes	2	8	3	7
Total	9	11	8	12
*p* value		*0.035*		0.325

Results at *p* ≤ 0.05 are shown in italics

Analysis of IL-34 showed that all tumors displayed mostly cytoplasmic immunopositivity for this protein (Fig. 1). Similar to M-CSF, the intratumoral staining pattern of IL-34 was irregular. Eight tumors showed moderate staining and the remaining 12 tumors showed strong staining. Statistical analysis of our scoring results showed no significant differences in IL-34 expression when it comes to tumor growth, cystic degeneration, CD-163 expression, or microvessel density.

Finally, cystic tumors showed a significantly higher degree of CD-163 expression compared to non-cystic tumors (*p* = 0.005).

## Discussion

Inflammation is an important feature of almost every type of neoplasm. The inflammatory process that takes places within tumors is often characterized by the abundance of tumor-associated macrophages [16]. The influx of these cells and their immunomodulating capacities allow the progression of tumor cells by stimulating processes such as of angiogenesis and cell survival [15]. M-CSF and IL-34 are cytokines that regulate macrophage recruitment, proliferation, and differentiation [24]. More specifically, M-CSF and recently also IL-34 are identified as important factors that polarize macrophages towards a protumoral M2 phenotype [22, 27]. High M-CSF expression and subsequent increased macrophage levels have been associated with disease progression and unfavorable outcome in several types of tumors [20, 21, 29] and similar findings are reported for IL-34 [26, 27].

Our earlier studies showed that VS can contain large quantities of TAM, and their presence seems to be related to tumor expansion. In this study, we attempted to find out if M-CSF and IL-34 are part of this process as well by comparing their expression pattern in progressive versus more indolent VS. A necessary condition to examine tumor tissue is that patients are surgically treated. One of the main indications for surgery is tumor growth. Even the group of slow-growing tumors showed a certain form of volumetric growth and therefore did not fully represent the truly indolent tumors. This unavoidable selection bias makes the identification of biological differences between indolent versus progressive variants of VS more challenging.

In this report, we demonstrate the presence of M-CSF and IL-34 in VS. The expression of M-CSF is higher in fast-growing VS compared to slower growing VS and, in accordance with its supposed function, the presence of M-CSF seems to be related to the expression of TAM within VS. Furthermore, the expression of M-CSF as well as the number of CD-163 positive macrophages appears to be higher in cystic tumors. We therefore postulate that intratumoral inflammation might also contribute to the pathogenesis of cystic degeneration. We could not demonstrate a relation between IL-34 expression and clinicopathologic characteristics of VS in our study group. Nevertheless, it should be noted that this protein was at least moderately expressed by all tumors, more than half of which even showed high immunopositivity. These relatively small intertumoral differences in expression of IL-34 together with the above mentioned selection bias may be a reason why we were unable to find a significant difference in the expression pattern of this protein.

It is important to note that the results of our comparisons are observations of association. There is always the possibility that these findings are epiphenomena of a larger biological growth process and therefore not directly related to each other. The fact remains that the results of this study seem to be in line with our earlier presented data on macrophage expression in VS. These current observations provide additional support for our hypothesis that the inflammatory microenvironment plays an essential role in the progression of these tumors. By modulating the characteristics of this microenvironment through the inhibition of M-CSF, and maybe IL-34, the progression of VS may in turn be decreased. For M-CSF, several clinical trials with inhibitors such as PLX3397 (pexidartinib) and RG7155 (emactuzumab) showed promising results in a variety of neoplasms [30, 31]. IL-34 might be a potential target as well but clinical evidence remains to be provided [32]. Before applying anti-*M-CSF* or anti-*IL-34* therapy in VS patients, in vivo schwannoma models would be very helpful to further elucidate the biological mechanisms that are involved in the associations observed in this study. Unfortunately, the current lack of sporadic vestibular schwannoma cell lines hampers such functional studies.

## References

[1] Evans DG, Moran A, King A, Saeed S, Gurusinghe N, Ramsden R (2005). Incidence of vestibular schwannoma and neurofibromatosis 2 in the north west of England over a 10-year period: higher incidence than previously thought. OtolNeurotol.

[2] Trofatter JA, MacCollin MM, Rutter JL, Murrell JR, Duyao MP, Parry DM, Eldridge R, Kley N, Menon AG, Pulaski K (1993). A novel moesin-, ezrin-, radixin-like gene is a candidate for the neurofibromatosis 2 tumor suppressor. Cell.

[3] Sainz J, Huynh DP, Figueroa K, Ragge NK, Baser ME, Pulst SM (1994). Mutations of the neurofibromatosis type 2 gene and lack of the gene product in vestibular schwannomas. Hum Mol Genet.

[4] Rouleau GA, Merel P, Lutchman M, Sanson M, Zucman J, Marineau C, Hoang-Xuan K, Demczuk S, Desmaze C, Plougastel B (1993). Alteration in a new gene encoding a putative membrane-organizing protein causes neuro-fibromatosis type 2. Nature.

[5] Stangerup SE, Caye-Thomasen P (2012). Epidemiology and natural history of vestibular schwannomas. Otolaryngol Clin N Am.

[6] Stangerup SE, Tos M, Thomsen J, Caye-Thomasen P (2010). True incidence of vestibular schwannoma?. Neurosurgery.

[7] Howitz MF, Johansen C, Tos M, Charabi S, Olsen JH (2000). Incidence of vestibular schwannoma in Denmark, 1977-1995. Am J Otol.

[8] Mautner VF, Nguyen R, Kutta H, Fuensterer C, Bokemeyer C, Hagel C, Friedrich RE, Panse J (2010). Bevacizumab induces regression of vestibular schwannomas in patients with neurofibromatosis type 2. Neuro Oncol.

[9] Plotkin SR, Halpin C, McKenna MJ, Loeffler JS, Batchelor TT, Barker FG (2010). Erlotinib for progressive vestibular schwannoma in neurofibromatosis 2 patients. Otol Neurotol.

[10] Wong HK, Lahdenranta J, Kamoun WS, Chan AW, McClatchey AI, Plotkin SR, Jain RK, di TE (2010). Anti-vascular endothelial growth factor therapies as a novel therapeutic approach to treating neurofibromatosis-related tumors. Cancer Res.

[11] Paldor I, Chen AS, Kaye AH (2016). Growth rate of vestibular schwannoma. J Clin Neurosci.

[12] de Vries M, Briaire-de BI, Malessy MJ, de Bruine SF, van der Mey AG, Hogendoorn PC (2013). Tumor-associated macrophages are related to volumetric growth of vestibular schwannomas. Otol Neurotol.

[13] de Vries M, Hogendoorn PC, Briaire-de BI, Malessy MJ, van der Mey AG (2012). Intratumoral hemorrhage, vessel density, and the inflammatory reaction contribute to volume increase of sporadic vestibular schwannomas. Virchows Arch.

[14] Mantovani A, Allavena P, Sica A, Balkwill F (2008). Cancer-related inflammation. Nature.

[15] Solinas G, Germano G, Mantovani A, Allavena P (2009). Tumor-associated macrophages (TAM) as major players of the cancer-related inflammation. J Leukoc Biol.

[16] Allen M, Louise JJ (2011). Jekyll and Hyde: the role of the microenvironment on the progression of cancer. J Pathol.

[17] Mantovani A, Marchesi F, Malesci A, Laghi L, Allavena P (2017). Tumour-associated macrophages as treatment targets in oncology. Nat Rev Clin Oncol.

[18] Lahmar Q, Keirsse J, Laoui D, Movahedi K, Van Overmeire E, Van Ginderachter JA (2016). Tissue-resident versus monocyte-derived macrophages in the tumor microenvironment. Biochim Biophys Acta.

[19] Svensson J, Jenmalm MC, Matussek A, Geffers R, Berg G, Ernerudh J (2011). Macrophages at the fetal-maternal interface express markers of alternative activation and are induced by M-CSF and IL-10. J Immunol.

[20] Ding J, Guo C, Hu P, Chen J, Liu Q, Wu X, Cao Y, Wu J (2016). CSF1 is involved in breast cancer progression through inducing monocyte differentiation and homing. Int J Oncol.

[21] Yang L, Wu Q, Xu L, Zhang W, Zhu Y, Liu H, Xu J, Gu J (2015). Increased expression of colony stimulating factor-1 is a predictor of poor prognosis in patients with clear-cell renal cell carcinoma. BMC Cancer.

[22] Kawamura K, Komohara Y, Takaishi K, Katabuchi H, Takeya M (2009). Detection of M2 macrophages and colony-stimulating factor 1 expression in serous and mucinous ovarian epithelial tumors. Pathol Int.

[23] Lin H, Lee E, Hestir K, Leo C, Huang M, Bosch E, Halenbeck R, Wu G, Zhou A, Behrens D, Hollenbaugh D, Linnemann T, Qin M, Wong J, Chu K, Doberstein SK, Williams LT (2008). Discovery of a cytokine and its receptor by functional screening of the extracellular proteome. Science (New York, NY).

[24] Nakamichi Y, Udagawa N, Takahashi N (2013). IL-34 and CSF-1: similarities and differences. J Bone Miner Metab.

[25] Segaliny AI, Brion R, Brulin B, Maillasson M, Charrier C, Teletchea S, Heymann D (2015). IL-34 and M-CSF form a novel heteromeric cytokine and regulate the M-CSF receptor activation and localization. Cytokine.

[26] Segaliny AI, Mohamadi A, Dizier B, Lokajczyk A, Brion R, Lanel R, Amiaud J, Charrier C, Boisson-Vidal C, Heymann D (2015). Interleukin-34 promotes tumor progression and metastatic process in osteosarcoma through induction of angiogenesis and macrophage recruitment. Int J Cancer.

[27] Baghdadi M, Wada H, Nakanishi S, Abe H, Han N, Putra WE, Endo D, Watari H, Sakuragi N, Hida Y, Kaga K, Miyagi Y, Yokose T, Takano A, Daigo Y, Seino KI (2016). Chemotherapy-induced IL34 enhances immunosuppression by tumor-associated macrophages and mediates survival of chemoresistant lung cancer cells. Cancer Res.

[28] Baranski Z, Booij TH, Cleton-Jansen AM, Price LS, van de Water B, Bovee JV, Hogendoorn PC, Danen EH (2015). Aven-mediated checkpoint kinase control regulates proliferation and resistance to chemotherapy in conventional osteosarcoma. J Pathol.

[29] Kluger HM, Dolled-Filhart M, Rodov S, Kacinski BM, Camp RL, Rimm DL (2004). Macrophage colony-stimulating factor-1 receptor expression is associated with poor outcome in breast cancer by large cohort tissue microarray analysis. Clin Cancer Res.

[30] Cannarile MA, Weisser M, Jacob W, Jegg AM, Ries CH, Ruttinger D (2017). Colony-stimulating factor 1 receptor (CSF1R) inhibitors in cancer therapy. J Immunother Cancer.

[31] Dammeijer F, Lievense LA, Kaijen-Lambers ME, van Nimwegen M, Bezemer K, Hegmans JP, van Hall T, Hendriks RW, Aerts JG (2017). Depletion of tumor-associated macrophages with a CSF-1R kinase inhibitor enhances antitumor immunity and survival induced by DC immunotherapy. Cancer Immunol Res.

[32] Zhou RP, Wu XS, Xie YY, Dai BB, Hu W, Ge JF, Chen FH (2016). Functions of interleukin-34 and its emerging association with rheumatoid arthritis. Immunology.

